# Ability of Self-Reported Frailty Components to Predict Incident Disability, Falls, and All-Cause Mortality: Results From a Population-Based Study of Older British Men

**DOI:** 10.1016/j.jamda.2016.08.020

**Published:** 2017-02-01

**Authors:** Efstathios Papachristou, S. Goya Wannamethee, Lucy T. Lennon, Olia Papacosta, Peter H. Whincup, Steve Iliffe, Sheena E. Ramsay

**Affiliations:** a Department of Primary Care and Population Health, UCL Medical School, London, United Kingdom; b Population Health Research Institute, St George's University of London, London, United Kingdom

**Keywords:** Frailty, mortality, disability, falls

## Abstract

**Background:**

Frailty is a state of increased vulnerability to disability, falls, and mortality. The Fried frailty phenotype includes assessments of grip strength and gait speed, which are complex or require objective measurements and are challenging in routine primary care practice. In this study, we aimed to develop a simple assessment tool based on self-reported information on the 5 Fried frailty components to identify older people at risk of incident disability, falls, and mortality.

**Methods:**

Analyses are based on a prospective cohort comprising older British men aged 71–92 years in 2010–2012. A follow-up questionnaire was completed in 2014. The discriminatory power for incident disability and falls was compared with the Fried frailty phenotype using receiver operating characteristic-area under the curve (ROC-AUC); for incident falls it was additionally compared with the FRAIL scale (fatigue, resistance, ambulation, illnesses, and loss of weight). Predictive ability for mortality was assessed using age-adjusted Cox proportional hazard models.

**Results:**

A model including self-reported measures of slow walking speed, low physical activity, and exhaustion had a significantly increased ROC-AUC [0.68, 95% confidence interval (CI) 0.63–0.72] for incident disability compared with the Fried frailty phenotype (0.63, 95% CI 0.59–0.68; *P* value of ΔAUC = .003). A second model including self-reported measures of slow walking speed, low physical activity, and weight loss had a higher ROC-AUC (0.64, 95% CI 0.59–0.68) for incident falls compared with the Fried frailty phenotype (0.57, 95% CI 0.53–0.61; *P* value of ΔAUC < .001) and the FRAIL scale (0.56, 95% CI 0.52–0.61; *P* value of ΔAUC = .001). This model was also associated with an increased risk of mortality (Harrell's C = 0.73, Somer's D = 0.45; linear trend *P* < .001) compared with the Fried phenotype (Harrell's C = 0.71; Somer's D = 0.42; linear trend *P* < .001) and the FRAIL scale (Harrell's C = 0.71, Somer's D = 0.42; linear trend *P* < .001).

**Conclusions:**

Self-reported information on the Fried frailty components had superior discriminatory and predictive ability compared with the Fried frailty phenotype for all the adverse outcomes considered and with the FRAIL scale for incident falls and mortality. These findings have important implications for developing interventions and health care policies as they offer a simple way to identify older people at risk of adverse outcomes associated with frailty.

Following a demographic shift observed in several countries, including the United Kingdom, the projected number of people aged ≥65 years is expected to rise by over 40% in the next 17 years.^1^ From a public health perspective, one of the major challenges of population aging is to reduce the morbidity and disability associated with increasing age. In the United Kingdom alone, 40% of those aged 65 years and older have a limiting longstanding illness,^1^ and 28%-35% experience falls every year.^2^

One of the most commonly recognized risk states for adverse outcomes in the older population is frailty, a clinical condition of increased vulnerability resulting from age-related declines in multiple physiological systems.^3^ Numerous prospective studies and meta-analyses have demonstrated significant associations between frailty status and increased risk of disability,^4^, ^5^ falls,^6^ and mortality.^4^, ^7^, ^8^, ^9^, ^10^, ^11^, ^12^ It is, therefore, recognized as one of the greatest challenges for health care professionals in countries with aging populations such as the United Kingdom.^13^ Frailty also offers potential for preventive management because it has been shown to be preventable or at least amenable to prevention of progression.^3^, ^14^ A review suggested that prescreening for frailty could serve as a 2-step approach to identify individuals who would benefit from further assessments, such as the comprehensive geriatric assessment (CGA).^15^ The CGA is a recommended component in the care of older patients and includes a detailed evaluation of the individual's functional status, physical health, psychological status including cognitive and affective status, and socioenvironmental factors.^16^ However, the CGA is time-consuming and, therefore, difficult to implement in routine community care. Consequently, there is a need to develop simple screening tools to identify old people who would benefit from a further detailed assessment followed by appropriate management to prevent adverse outcomes of frailty.^15^

Two models of frailty, the frailty phenotype and the frailty index, have provided the conceptual basis to measure frailty. The Fried frailty phenotype comprises weight loss, physical inactivity, slow walking speed, low grip strength, and exhaustion.^7^ The frailty index is a cumulative score of a number of symptoms, signs, disease, abnormal laboratory results, and disability.^17^ Currently, more than 27 indices aiming to screen for frailty status have been described in the literature.^12^, ^13^, ^14^, ^18^, ^19^ The majority of these scales are extensive, for example the 25-item Tilburg Frailty Indicator,^20^ the 15-item Groningen frailty indicator,^21^ the 70-item frailty index,^22^, ^23^ and the 11-item Edmonton Frail scale.^24^, ^25^ Commonly, they also necessitate objective measures of grip strength and/or gait speed (eg, the Fried frailty component^7^ or the Survey of health, ageing, and retirement in Europe-frailty instrument [SHARE-FI]^26^), and are, therefore, challenging in routine primary care practice. Attempts to develop a simple screening tool for frailty, such as the 5-item FRAIL scale, comprising Fatigue, Resistance, Ambulation, Illnesses, and Loss of weight,^14^, ^27^, ^28^, ^29^ have shown promising results in terms of their ability to detect frailty among middle-aged and older populations and to predict incident functional loss and mortality.^27^, ^30^

This study in a community-dwelling cohort of older British men aimed to investigate the ability of simple self-reported measures of the Fried frailty components, including weight loss, physical inactivity, slow walking speed, low grip strength, and exhaustion to predict incident disability, falls, and mortality over a 3-year follow-up period. The discriminative and predictive ability of models including up to 3 subjective measures was compared with that of the Fried frailty phenotype and with the simpler FRAIL scale.

## Methods

Data for this study are based on the British Regional Heart Study (BRHS), a prospective cohort study comprising a socially and geographically representative sample of 7735 men aged 40–59 years from 1 general practice in each of 24 towns representing all major British regions and who were initially examined in 1978–1980.^31^ Surviving study members aged 71–92 years (n = 3137) were invited to attend a 30-year reexamination in 2010–2012, of whom 2137 completed a questionnaire (68% response rate), and 1722 attended a physical examination (55% response rate).^32^ In 2014, a follow-up postal questionnaire was sent to the cohort and was completed by 1655 participants (64% response rate). In total, 1198 study participants had complete data during the 30-year reexamination and the follow-up questionnaire. Ethical approval was provided by the relevant research ethics committees. All men provided written informed consent to the investigations, which were carried out in accordance with the Declaration of Helsinki.

Physical examination of participants at age 71–92 years involved anthropometric (height, weight) and physical performance (gait speed, grip strength) assessments, as well as a lung function test. Height was measured with a Harpenden stadiometer to the last complete 0.1 cm and weight with a Tanita MA-418-BC body composition analyzer (Tanita, Tokyo, Japan). Body mass index was calculated as weight/(height)^2^ (kg/m^2^). Grip strength (in kilograms) was measured with a Jamar hydraulic hand dynamometer (Model J00105, Lafayette Instrument Europe, Leicester, UK). Walking speed was defined as the time taken, in seconds, to walk 3 meters at normal walking pace. If walking speed was unavailable, self-reported information of slow walking pace (“being unable to walk more than a few steps or <200 yards or difficulty walking across a room”) was used. Three measurements were taken for each hand, and the best of 6 readings was used for the analysis. Forced expiratory volume in 1 second and forced vital capacity were measured using a Vitalograph compact II spirometer (Vitalograph Ltd, Buckingham, UK) with the participant seated.

Subjective assessments of walking speed, grip strength, weight loss, exhaustion, and physical activity were derived from the questionnaire completed in 2010–2012. They included single-item questions on self-reported (1) inability to grip with hands (eg, opening a jam jar); (2) decrease of weight in the last 4 years; (3) slow walking pace; (4) not feeling full of energy; and (5) being less or much less active compared with a man who spends 2 hours on most days on activities such as walking, gardening, household chores, or do-it-yourself projects.

Additional baseline sample characteristics considered included social class, smoking status, and alcohol consumption. For alcohol intake, the men were classified into 5 groups—none, occasional, light, moderate, and heavy. Heavy drinking was defined as drinking >6 units (1 UK unit = 10 g) of alcohol daily or on most days. Men were also classified in 4 groups according to their smoking habits as current smokers, ex-smokers who gave up smoking before or after 1983, and those who never smoked.

Frailty and prefrailty status based on the Fried frailty phenotype and the FRAIL scale was derived for participants attending the 30-year reexamination of the BRHS using information drawn from the questionnaire and the physical examination.^33^ The Fried frailty phenotype components included (1) unintentional weight loss defined as ≥ 5% decrease in self- reported weight, which was reported to be unintentional; (2) weakness defined as being in the lowest quintile of the distribution for grip strength; (3) low physical activity was assessed using self-report questions on being less or much less active than an average man, or participating in active sport and endurance activities; (4) exhaustion was defined as participants reporting not to be feeling full of energy; and (5) slow walking speed was defined as being in the lowest quintile of the distribution of walking speed. Scores on the FRAIL scale were computed using information on exhaustion, resistance, ambulation, illnesses, and weight loss. Measurements of exhaustion and weight loss were the same for the Fried frailty phenotype and the FRAIL scale. Ambulation was computed using information on the ability to walk more than 200 yards. Resistance was based on information on the ability to climb a flight of 12 stairs. Participants were considered to have multiple illnesses when they reported having a history of at least 5 out of 11 total illnesses, including hypertension, diabetes, cancer, chronic lung disease, heart attack, heart failure, angina, asthma, arthritis, stroke, and kidney disease. Participants were considered to have a positive history of chronic lung disease when they reported being prescribed bronchodilators (British National Formulary code 3.1) and had forced expiratory volume in 1 second/forced vital capacity <0.70.^34^ For both, the Fried frailty phenotype and the FRAIL scale, presence of 3 or more components was defined as frailty, and presence of 1 or 2 as prefrailty.

Outcome measures of disability and falls were derived from the questionnaire completed in 2014. Information on all-cause mortality was obtained from death certificates. Disability was defined as mobility limitations and measured using information on difficulties going up or down stairs, or walking 400 yards. Falls were assessed using self-reported information on whether participants experienced falls during the previous year.

### Statistical Analyses

We calculated age adjusted odds ratios to estimate the relationship of the Fried frailty components and self-reported (subjective) frailty questions with incident disability and falls over the 3-year follow-up period. Cox proportional hazard models were used to obtain age-adjusted hazard ratios (HR) for mortality. We used receiver operating characteristic-area under the curve (ROC-AUC) to assess the discriminatory ability of the subjective frailty measures for incident disability and falls. Using the *roccomp* command in Stata we compared the ROC-AUC obtained by the objectively assessed Fried frailty phenotype components and the subjective measures. The subjective measures that produced ROC-AUC comparable to the Fried frailty phenotype for incident falls and disability were used to create composite indices with up to 3 variables. We assessed the ROC-AUC of these composite indices and evaluated the improvement in their ability to reclassify falls and disabilities by calculating the integrated discrimination index (IDI). The models that best predicted disability and falls were tested for prediction of mortality using age-adjusted Cox proportional hazard models and were compared with the Fried frailty phenotype using the Harrell's-C statistic. In addition, we compared our models with the FRAIL scale for falls and mortality, but not for incident disability (mobility limitation), as this was part of the FRAIL scale. All analyses were performed using Stata/SE 14 (Stata Corp, College Station, TX).

## Results

Of 1622 participants who attended the physical examination in 2010–2012, complete data on falls or disability in the follow-up questionnaire in 2014 were available for 1198 study members. Of those, 380 (32%) were classified as nonfrail, 652 (54%) as prefrail, and 166 (14%) as frail based on the Fried frailty phenotype. In addition, 521 (43%) were classified as nonfrail, 580 (48%) as prefrail, and 97 (8%) as frail according to the FRAIL scale. The characteristics of the baseline sample are summarized in Table 1. Over the 3-year follow-up period (mean = 2.96 years, standard deviation = 0.52), 128 (15%) participants developed incident disability, and 163 (16%) reported at least 1 fall within the previous 12 months. A total of 83 deaths occurred (5%) during this follow-up period with a mean survival time of 1.17 years (mean = 1.17 years, standard deviation = 1.16).

Table 2 presents the associations of subjectively and objectively assessed frailty components with incident disability, falls, and all-cause mortality over the 3-year follow-up period. After adjustments for age, both objectively and subjectively assessed physical inactivity and slow walking speed were significantly associated with incident disability, falls and all-cause mortality. Objectively assessed weight loss was significantly associated with a higher risk for mortality only; in contrast, subjectively assessed weight loss was additionally associated with a higher risk for incident falls. Being in the bottom quintile of physical assessments of grip strength was not significantly predictive of any of the considered outcomes. However, self-reported inability to grip with hands was significantly associated with incident disability and falls. Finally, exhaustion was significantly associated with incident disability, but not incident falls or mortality. Based on the Fried frailty phenotype, prefrail and frail participants were at a higher risk for incident disability and mortality, but only frail individuals were at a significantly higher risk for incident falls.

Table 3 presents the discriminative ability of the Fried frailty phenotype and the subjective frailty components for incident disability over the 3-year follow-up period. Results of the ROC tests showed moderate ability of the Fried frailty phenotype to discriminate between participants with and without incident disability [AUC = 0.63, 95% confidence interval (CI) 0.59–0.68]. Of the subjectively measured frailty components, walking speed, physical inactivity, and exhaustion had comparable AUC values to the one obtained by the Fried frailty phenotype, while weight loss and grip strength showed significantly reduced discriminatory ability. Therefore, subjectively measures of walking speed, physical activity, and exhaustion were used to identify models predictive of incident disability. Models including any of these 2 subjective frailty components did not have an appreciably higher AUC than the one obtained for the Fried frailty phenotype (all *P* values > .05). However, the model including slow walking speed, physical inactivity, and exhaustion, showed a significant improvement in the discriminative ability for incident disability (AUC = 0.68, 95% CI 0.63–0.72 for model D in Table 3) in comparison to the Fried frailty phenotype (*P* = .01). In addition, the significant IDI (IDI = 1%; *P* = .03) also suggested improvement in the reclassification of cases with incident disability compared with the model, which included slow walking speed and physical inactivity only.

Table 4 summarizes the discriminative ability of the Fried frailty phenotype, the FRAIL scale, and the subjective frailty components for incident falls over the 3-year follow-up period. Compared with the ROC-AUC of the Fried frailty phenotype for incident falls (AUC = 0.57, 95% CI 0.53–0.61), subjectively assessed gait speed, physical activity, weight loss, and grip strength had comparable AUC values, whereas the ROC-AUC of exhaustion was significantly lower. Therefore, the self-reported frailty components used to build a model for prediction of incident falls included slow walking speed, physical inactivity, weight loss, and low grip strength. Of all 2-variable models considered, the ones including slow walking speed and physical inactivity (model A in Table 4), or slow walking speed and weight loss (model B in Table 4) had a significantly increased ROC-AUC compared with the one obtained by the Fried frailty phenotype. Next, we tested models including combinations of up to 3 self-reported frailty components. The highest ROC-AUC was obtained for a model including slow walking speed, physical inactivity, and weight loss (AUC = 0.64, 95% CI 0.59–0.68; *P* value of ΔAUC compared with the Fried frailty status <.001; model G in Table 4). An additional model including slow walking speed, physical inactivity, and low grip strength (AUC = 0.62, 95% CI 0.57–0.66; model H in Table 4) also had a significantly increased AUC-value (AUC = 0.62, 95% CI 0.57–0.66) compared with the one obtained by the Fried frailty phenotype (*P* = .01) while also retaining a significant IDI value (IDI = 0.4%; *P* = .05). In addition, both models best predicting incident falls had a significantly increased AUC-value compared with the one obtained by the FRAIL scale (AUC = 0.56, 95% CI 0.52–0.61; both *P* values of ΔAUC ≤.01).

The model that best predicted disability (self-reported slow walking speed + physical inactivity + exhaustion) was significantly associated with mortality (HR = 1.61, 95% CI 1.31–1.98). However, the associated Harrell's C and Somer's D statistics (Harrell's C = 0.69; Somer's D = 0.38) were lower than that obtained by the Fried frailty phenotype (HR = 2.81, 95% CI 1.96–4.05; Harrell's C = 0.71; Somer's D = 0.42) and the FRAIL scale (HR = 2.86, 95% CI 2.04–4.03; Harrell's C = 0.71; Somer's D = 0.42) suggesting reduced predictive power. The first of the 2 models that best predicted falls (self-reported slow walking speed + physical inactivity + weight loss) was significantly associated with an increased risk for mortality (HR = 2.08, 95% CI 1.68–2.59) and had also increased predictive power (Harrell's C = 0.73; Somer's D = 0.45) compared with the Fried frailty phenotype and the FRAIL scale. In contrast, the second model that was predictive of incident falls (self-reported slow walking speed + physical inactivity + low grip strength) also had an increased risk for mortality (HR = 1.65, 95% CI 1.34–2.02), but its predictive ability (Harrell's C = 0.69; Somer's D = 0.39) was lower than that of the Fried frailty phenotype and the FRAIL scale.

## Discussion

In this study, we demonstrate that self-reported information on walking speed, physical activity, exhaustion, and weight loss can be used to identify older people at risk of incident disability, falls, and all-cause mortality, which are known adverse outcomes of frailty. When combined, these simple and subjective assessments of frailty components had superior discriminatory and predictive ability compared with the Fried frailty phenotype for all the adverse outcomes considered and with the FRAIL scale for incident falls and mortality. Such measures in community care can offer a simple way of identifying vulnerable older individuals who would benefit from a further intensive assessment such as a CGA^15^ without having to rely on more complex and time-consuming assessments.

In particular, single self-reported items on walking speed and physical activity were predictive of incident disability, falls, and mortality, and their individual and combined discriminative ability for these outcomes was comparable to that of the Fried frailty phenotype. Recent reviews and meta-analyses provide additional evidence that reduced gait speed 35, 36, 37 and physical inactivity^38^, ^39^ are key components of at-risk states for disability and falls. Self-reported weight-loss was predictive of incident falls and mortality, whereas exhaustion was associated only with incident disability. Unlike other studies, objectively assessed grip strength was not a significant independent predictor of any of the outcomes considered in our study.^40^ Yet, self-reported grip strength was significantly associated with incident disability and falls.

When self-reported information of exhaustion was combined with self-reported walking speed and physical inactivity, the discriminative ability for incident disability increased significantly (ROC-AUC = 0.68). This finding is in line with results of a study on community-dwelling older adults, which has demonstrated the association of fatigue with mobility-related disability.^41^ Moreover, the ability of subjective walking speed and physical activity to predict incident falls increased substantially on including information on self-reported weight loss (ROC-AUC = 0.64). The ROC-values observed for incident falls and disability using these self-reported frailty measures were significantly higher compared with the one obtained for the Fried frailty phenotype in this sample. They are also comparable with the absolute AUC values for falls and disability reported in a cohort study of older men that examined both the Fried frailty phenotype and a simpler index comprising weight loss, inability to rise from a chair, and poor energy.^42^ Combined self-reported information on subjective walking speed, physical activity, and weight loss was also significantly better in predicting incident falls and mortality compared with the scores of the FRAIL scale. Nonetheless, the FRAIL scale yielded discriminative and predictive ability for incident falls and mortality that was comparable to that of the Fried frailty phenotype; this also provides evidence for the adequacy of simpler screening tools that do not necessitate complex objective assessments.

One of the main strengths of this study is that it is based on a large prospective population-based study representative of older British men. The cohort has a high follow-up rate of 98% and has enabled very low attrition rates. However, the response rate for the baseline assessment in this study was 55%, and, therefore, the issue of survival bias cannot be overlooked. Frail individuals in particular are likely to have died at a higher rate.^12^ Of the 2137 participants who attended the 30-year reexamination of the BRHS, 1722 attended the physical examination, which presents an additional selection bias. Moreover, the BRHS includes only white European men and, therefore, generalizability to women and other ethnic minorities is limited.

## Conclusions

This study provides evidence that single self-reported items on weight loss, slow walking speed, physical inactivity, and exhaustion are significant predictors of adverse outcomes associated with frailty in older people, including incident disability, falls, and all-cause mortality. When combined, these self-reported items can provide a better risk stratification for these adverse outcomes than the Fried frailty phenotype, which necessitates standardized objective assessments. Objective assessments required to assess the Fried frailty phenotype and other frailty indices can be challenging in routine community care because they are more time-consuming. Moreover, the discriminative and predictive ability of self-reported frailty components for incident falls and mortality in our sample was superior to that obtained by the FRAIL scale, which further supports the robustness of our findings. The results of this study have important implications for health care policies as they offer a very simple way to identify people at risk of adverse outcomes associated with frailty. The findings of this study could also form the basis of studies developing interventions to target older individuals who would benefit from further detailed assessments, or further management aiming to prevent disabilities and falls in older age.

## Figures and Tables

**Table 1:** Demographic and Lifestyle Factors According to Frailty Status in a Population-Based Sample of 1198 British Men Age 71–92 Years in 2010–2012

Variables	Nonfrail (n = 380; 32%)	Prefrail (n = 652; 54%)	Frail (n = 166; 14%)	Total (n = 1198)	*P* Value∗
Age (years)	76.66 (3.62)	78.27 (4.41)	79.69 (4.88)	77.95 (4.36)	<.001
BMI	26.69 (3.45)	27.18 (3.55)	28.32 (4.39)	27.18 (3.68)	<.001
Manual social class, n (%)	163 (44%)	277 (44%)	77 (48%)	517 (44%)	.55
Smoking status/never smoked, n (%)	163 (43%)	244 (37%)	55 (33%)	462 (39%)	.07
Moderate/heavy alcohol consumption, n (%)	7 (2%)	16 (3%)	4 (2%)	27 (2%)	.79

BMI, body mass index.

Continuous variables as shown as mean (standard deviation).

∗ *P* value of respective χ^2^ test or 1-way analysis of variance (ANOVA).

**Table 2:** Odds Ratios (OR) and hazard ratios (HR) (95% CIs) for Incident Falls, Disability, and All-Cause Mortality According to Frailty Components and Self-Report Frailty Measures Over a 3-Year Follow-Up Period in a Cohort of 1198 British Men Aged 71–92 Years

	Incident Disability (n = 138; 15%)	Incident Falls (n = 163; 16%)	Mortality (n = 83; 5%)
	n (%)| OR (95% CI)	n (%)| OR (95% CI)	n (%)| HR (95% CI)
**Gait speed**			
Fried frailty component			
Lowest quintile of walking speed	30 (22%) **3.24 (1.97–5.34)**	38 (23%) **1.99 (1.30–3.04)**	39 (47%) **2.90 (1.84–4.56)**
Single self-report item			
Slow walking pace	41 (30%) **3.97 (2.53–6.21)**	55 (34%) **2.27 (1.56–3.32)**	45 (55%) **2.98 (1.91–4.66)**
**Physical Activity**			
Fried frailty component			
Physically Inactive	48 (35%) **2.57 (1.72–3.83)**	60 (37%) **1.80 (1.26–2.58)**	44 (53%) **2.17 (1.40–3.36)**
Single self-report item			
Less active or much less active in comparison to other men	43 (32%) **2.63 (1.73–4.00)**	55 (34%) **1.83 (1.26–2.64)**	41 (49%) **2.18 (1.41–3.37)**
**Weight loss**			
Fried frailty component			
≥5% unintentional weight loss	15 (11%) 1.33 (0.72–2.45)	20 (12%) 1.50 (0.88–2.57)	29 (35%) **3.88 (2.44–6.16)**
Single self-report item			
Weight loss in the last four years	31 (22%) 1.44 (0.92–2.26)	43 (27%) **1.84 (1.24–2.74)**	36 (44%) **3.14 (2.02–4.87)**
**Grip strength**			
Fried frailty component			
Bottom quintile of grip strength	30 (22%) 1.18 (0.75–1.86)	39 (24%) 1.48 (0.98–2.22)	28 (34%) 1.22 (0.76–1.97)
Single self-report item			
Difficulty or unable to grip with hands	24 (17%) **1.93 (1.16–3.22)**	33 (21%) **1.73 (1.11–2.70)**	24 (30%) 1.51 (0.93–2.46)
**Exhaustion**			
Reporting low energy	85 (62%) **2.32 (1.60–3.38)**	81 (50%) 1.04 (0.74–1.46)	56 (67%) 1.56 (0.98–2.49)
**Fried frailty phenotype**			
Prefrail	81 (59%) **1.85 (1.19–2.89)**	94 (58%) 1.36 (0.91–2.01)	37 (45%) **2.64 (1.11–6.28)**
Frail	26 (19%) **6.19 (3.29–11.65)**	27 (17%) **2.06 (1.18–3.59)**	40 (48%) **7.60 (3.15–18.31)**

Bold indicates *P* < .05; OR and HR are age-adjusted.

**Table 3:** Discriminative Ability of Models With up to 3 Self-Reported Frailty Components Compared With Fried Frailty Phenotype for Incident Disability Over a 3-Year Follow-Up Period in a Cohort of 1198 British Men Aged 71–92 Years

	Incident Disability		
	ROC-AUC (95% CI)	*P* Value ΔAUC∗	IDI (*P* Value†)
**Fried frailty phenotype**	0.63 (0.59–0.68)	NA	NA
**Self-reported single-variable models**			
Slow walking speed	0.61 (0.57–0.65)	**.44**	NA
Exhaustion	0.61 (0.57–0.66)	**.25**	NA
Physically inactive	0.59 (0.56–0.64)	**.09**	NA
Low grip strength	0.54 (0.51–0.58)	<.001	NA
Weight loss	0.54 (0.50–0.57)	<.001	NA
**Self-reported 2-variable models**			
Model A: Slow walking speed (ref) + physically inactive	0.65 (0.60–0.69)	.45	**.01 (.03)**
Model B: Slow walking speed (ref) + exhaustion	0.66 (0.62–0.71)	.12	**.01 (.02)**
Model C: Physically inactive (ref) + exhaustion	0.64 (0.59–0.69)	.58	**.01 (.001)**
**Self-reported 3-variable models**			
Model D: Model A (ref) + exhaustion	0.68 (0.63–0.72)	**.01**	**.01 (.03)**

NA, not applicable.

Bold indicates comparable or significantly increased discriminatory ability compared with the Fried frailty phenotype.

∗ Pairwise comparison of ROC-AUC of each model and the one obtained by the Fried frailty phenotype.

† *P* value of reclassification improvement over the reference category.

**Table 4:** Discriminative Ability of Models With up to 3 Self-Reported Fried Frailty Components Compared With Fried Frailty Phenotype and the FRAIL Scale for Incident Falls Over a 3-Year Follow-Up Period in a Cohort of 1198 British Men Aged 71–92 Years

	Incident Falls			
	ROC-AUC (95% CI)	*P* Value ΔAUC∗	*P* Value ΔAUC†	IDI (*P* Value‡)
**Fried frailty phenotype**	0.57 (0.53–0.61)	NA	NA	NA
**FRAIL scale**	0.56 (0.52–0.61)	NA	NA	NA
**Self-reported single-variable models**				
Slow walking speed	0.58 (0.55–0.62)	**.40**	**.28**	NA
Physically inactive	0.57 (0.53–0.61)	**.96**	**.70**	NA
Low grip strength	0.54 (0.51–0.58)	**.25**	**.40**	NA
Weight loss	0.55 (0.52–0.59)	**.61**	**.74**	NA
Exhaustion	0.52 (0.47–0.56)	.004	.001	NA
**Self-reported 2-variable models**				
Model A: Slow walking speed (ref) + physically inactive	0.61 (0.56–0.65)	**.04**	**.04**	.002 (.32)
Model B: Slow walking speed (ref) + weight loss	0.62 (0.57–0.66)	**.04**	**.03**	.006 (.08)
Model C: Slow walking speed (ref) + low grip strength	0.60 (0.56–0.64)	.14	.09	**.005 (.05)**
Model D: Physically inactive (ref) + weight loss	0.60 (0.56–0.65)	.14	.08	**.007 (.03)**
Model E: Physically inactive (ref) + low grip strength	0.59 (0.54–0.63)	.43	.28	**.005 (.04)**
Model F: Weight loss (ref) + low grip strength	0.59 (0.55–0.63)	.38	.24	.006 (.06)
**Self-reported 3-variable models**				
Model G: Model A (ref) + weight loss	0.64 (0.59–0.68)	**<.001**	**.001**	.005 (.09)
Model H: Model A (ref) + low grip strength	0.62 (0.57–0.66)	**.01**	**.01**	**.004 (.05)**
Model I: Model D (ref) + low grip strength	0.62 (0.58–0.66)	**.02**	**.01**	.004 (.09)

NA, not applicable.

Bold indicates comparable or significantly increased discriminatory ability.

∗ Pairwise comparison of ROC-AUC of each model and the one obtained by the Fried frailty phenotype.

† Pairwise comparison of ROC-AUC of each model and the one obtained by the FRAIL scale.

‡ *P* value of reclassification improvement over the reference category.
